# Potential of Mobile Health Technology to Reduce Health Disparities in Underserved Communities

**DOI:** 10.5811/westjem.2019.6.41911

**Published:** 2019-08-06

**Authors:** Tara van Veen, Sophia Binz, Meri Muminovic, Kaleem Chaudhry, Katie Rose, Sean Calo, Jo-Ann Rammal, John France, Joseph B. Miller

**Affiliations:** * Henry Ford Hospital, Department of Emergency Medicine, Henry Ford Hospital, Detroit, Michigan; † Wayne State University School of Medicine, Detroit, Michigan; ‡ Henry Ford Hospital, Department of Internal Medicine, Detroit, Michigan; § Central Michigan University School of Medicine, Mount Pleasant, Michigan; ¶ Inova Fairfax Department of Surgery, Fairfax, Virginia

## Abstract

**Introduction:**

Mobile health (mHealth) has the potential to change how patients make healthcare decisions. We sought to determine the readiness to use mHealth technology in underserved communities.

**Methods:**

We conducted a cross-sectional survey of patients presenting with low-acuity complaints to an urban emergency department (ED) with an underserved population. Patients over the age of two who presented with low-acuity complaints were included. We conducted structured interview with each patient or parent (for minors) about willingness to use mHealth tools for guidance. Analysis included descriptive statistics and univariate analysis based on age and gender.

**Results:**

Of 560 patients included in the survey, 80% were adults, 64% female, and 90% Black. The mean age was 28 ± 9 years for adults and 9 ± 5 years for children. One-third of patients reported no primary care physician, and 55% reported no access to a nurse or clinician for medical advice. Adults were less likely to have access to phone consultation than parents of children (odds ratio [OR] 0.49, 95% confidence interval [CI], 0.32 – 0.74), as were males compared to females (OR 0.52, 95% CI, 0.37–0.74). Most patients (96%) reported cellular internet access. Two-thirds of patients reported using online references. When asked how they would behave if an mHealth tool advised them that their current health problem was low risk, 69% of patients responded that they would seek care in an outpatient clinic instead of the ED (30%), stay home and not seek urgent medical care (28%), or use telehealth (11%).

**Conclusion:**

In this urban community we found a large capacity and willingness to use mHealth technology in medical triage.

## INTRODUCTION

Minority and low-income patients have high levels of cell phone and mobile internet use.^1^,^2^ Mobile health (mHealth) has the potential to enhance healthcare for underserved populations with limited access to traditional healthcare resources.^3^ Emergency departments (ED) are increasingly being used as a safety net for underserved populations with health conditions that could be treated in the primary care setting.^4^ Development of high-quality mHealth tools to connect these underserved populations to medical advice could reduce ED utilization for low-acuity complaints. While the potential exists to decrease such disparities in healthcare access, the willingness of these patients to use mHealth is not well understood.^3^ Hence, our goal was to determine healthcare access and readiness to engage with mHealth technology among patients using an urban ED for low-acuity complaints.

## METHODS

### Study Design, Setting, and Selection of Participants

This study was a cross-sectional survey in an academic ED (>100,000 annual patient volume) with a large minority and low-income population. Enrollment occurred from June 2016 to January 2017 in Detroit, Michigan. At that time, the median income was $26,249 and 39.4% of the population was living below the federal poverty level. Patients were approached based on chief complaint. Research associates obtained informed consent from patients that met inclusion criteria. For children (<18 years), parents provided informed consent and completed the survey. Investigators also collected relevant clinical and demographic information from the electronic health record.

We included patients and parents of children presenting to the low-acuity section of the ED with chief complaints of sore throat, cough and congestion, non-traumatic headache, and symptoms of sexually transmitted infections. Exclusion criteria included patients <2 years and >50 years old, severe illness with expected hospital admission, and inability to provide informed consent.

### Measures

The brief, 15-item, survey instrument focused on patient interest in mHealth and healthcare access (Appendix 1). We developed and refined items based on interviews with ED patients. Research associates administered surveys in person, and patients either completed a written paper form or verbally responded to survey questions based on their preference. We used REDCap electronic data capture tools to compile and code all survey data.

### Outcomes and Data Analysis

The primary outcome was a descriptive assessment of healthcare access and engagement in mHealth technology. A formal sample size calculation was not performed. Analysis included descriptive statistics and univariate analysis with SAS 9.4 (Cary, NC). We used logistic regression to determine differences in mHealth use and engagement based on age and gender. We report odds ratios (OR) with 95% confidence intervals (CI). For the purpose of comparing age, we divided participants into millennials (birth year ≥ 1982) and non-millennials. The local institutional review board approved the study.

**Population Health Research Capsule**

What do we already know about this issue?

*Mobile health (mHealth) technology has the potential to decrease ED visits and reduce cost in underserved communities*.

What was the research question?

*We sought to determine the readiness of patients in underserved communities to use mHealth technology*.

What was the major finding of the study?

*For medical triage, there is significant capacity and willingness to use mHealth technology*.

How does this improve population health?

*This study identifies mHealth as an avenue to help patients in underserved communities better align their medical problems with appropriate care*.

## RESULTS

### Characteristics of Study Subjects

A total of 560 patients participated in the study. Most of the patients were adults (449, 80%) and 360 (64%) were female. African Americans represented 496 (89%) of participants, Caucasians 29 (5%), and other races 35 (6%). The mean age was 28±9 years among adults and 9±5 years among children. More parents that completed questionnaires were female (65%) compared to male (46%). Serious comorbidities were uncommon but included 109 (24%) patients with asthma, hypertension 79 (18%), and diabetes 22 (5%).

### Access to Care

One-third of study participants denied having a primary care doctor (Table 1). The majority of patients (55%) also denied phone access to a nurse or clinician for advice. Female participants reported higher access to primary care than men. Adults were less likely to have telephone access for healthcare advice compared to parents of children (OR 0.49, 95% CI, 0.32 – 0.74). Males were less likely than females to have access to healthcare advice (OR 0.52, 95% CI, 0.37 – 0.74). Only 342 (61%) of patients reported computer internet access, but 538 participants reported access to mobile internet use (96%). There was no difference in mobile internet capacity between gender and age. Participants reported seeking medical advice from internet resources as often as friends or family members. The most commonly used internet resources were Google (66%) and WebMD (14%).

### Willingness to Use Mobile Health

Most participants (92%) indicated that they would use a mHealth application to assist in triaging their current condition (Table 2). Among those who indicated they would be unlikely to use a mHealth application, the most common reasons were having ready access to a physician; no access to a reliable phone; or a preference for an individual assessment. The majority of patients indicated that they would avoid an ED visit if the mHealth tool suggested that their current health issue was low risk for a health emergency (Table 2).

## DISCUSSION

Our results show that access to primary care providers for both clinic visits and medical triage advice is poor in this underserved population. Our study found that women had increased medical access, which is consistent with the current literature.^5^ Nevertheless, this community has high rates of mobile phone use, internet capability, and patients who use the internet to research their symptoms. These results contrast to a 2012 study that found that only 21% of an underserved population used the internet for health information compared to 61% of the general population.^6^ A 2015 study found 71% of ED patients had smartphones and 44% of smartphone users had health applications.^7^ Our study consisted of a much larger cohort of ED patients and found that nearly every participant had access to internet cell phone use. This difference likely reflects a younger cohort and increasing access to mobile phones.

Our results show that ED patients with low-acuity complaints are willing to use a validated application to assist in triage for their condition. The vast majority of patients said they would “definitely” or “probably” use a validated application. Additionally, nearly 70% of participants were willing to choose non-emergent healthcare for their current condition if they had access to a reliable mHealth tool that indicated low risk of medical emergency.

A validated mHealth tool has the potential to assist in making important decisions as to where and when to seek care. Particularly in underserved populations, such a tool has the potential to decrease ED visits and lower healthcare costs.^8^ Despite this potential, the willingness of underserved populations to use mHealth requires further study. Participants who responded to our hypothetical scenario were already under the care of a healthcare provider and may have been reassured answering in the manner they did than if they were to consider such questions prior to coming to the ED. We did not apply a triage mHealth tool in practice prior to arrival. Furthermore, some existing data suggests that patients make inconsistent decisions based on mHealth data.^7^ Low health literacy may also be a factor that prevents patients from adequately interacting with mHealth tools to make informed decisions.^9^

Whether mHealth tools are ready to address these disparities remains to be seen. Symptom checkers have proliferated through web-based or app-based mHealth resources. These tools typically use algorithms (often enabled by artificial intelligence) to help patients with self-diagnosis or self-triage. Nevertheless, validation of these tools is lacking. In one study testing whether 23 different symptom checkers could provide accurate triage, correct triage of non-emergent cases was relatively poor (55%).^10^ The authors note that symptom checkers are generally risk adverse and err toward recommending emergent care more often than is necessary. Nurse-staffed telephone triage lines may also err toward recommending emergent care more often than is necessary. There is evidence that physician-based telemedicine triage tools are equivalent to in-person physician triage tools.^11^ However, whether improved mHealth algorithms can outperform nurse-staffed triage remains to be seen.

## LIMITATIONS

There are several notable limitations. First, results from this convenience sample of eligible, low-acuity patients may not translate to a broader group of ED patients, non-English speakers, and other underserved populations. We targeted a population of young patients and parents in this study. Older ED patients likely experience access to care and use of mHealth differently. Second, we designed and refined our survey instrument based on limited existing literature and patient response. The instrument did not undergo rigorous validation prior to data collection. Finally, it is notable that primary care access remains a barrier. Even though patients may be willing to use mHealth tools for triage purposes, these tools may reduce low-acuity ED visits if primary care or urgent care access is poor.

## CONCLUSION

In an urban, low-income community of young adults and parents of children, there is a high degree of capacity and willingness to implement mHealth technology to guide medical triage. In settings where adequate healthcare access may be lacking, these results highlight the potential for mHealth to reduce disparities related to medical triage.

## Supplementary Information



## Figures and Tables

**Table 1:** Access to healthcare and internet services.

		All, n (%)	Male, n (%)	Female, n (%)	p-value
Provider Access	Primary care provider	372 (66)	111 (56)	261 (73)	<0.001
	Clinic/nurse phone line*	253 (45)	70 (35)	183 (50)	<0.001
Online Acess	Phone applications	414 (76)	151 (77)	263 (75)	0.693
	Computer internet access	342 (61)	120 (60)	222 (62)	0.698
	Phone internet access	538 (96)	190 (95)	348 (97)	0.331
Primary Health Resources	Friends or family	375 (67)	127 (64)	248 (69)	0.194
	Medical reference book	205 (37)	67 (34)	138 (38)	0.255
	Online medical search	382 (68)	126 (63)	256 (71)	0.048

* Access to nurse or clinician after-hours phone line to call for medical advice.

**Table 2:** Response to mobile health tool.

	Response, n (%)			
Willingness to use mHealth tool to triage current condition	Definitely 314 (56)	Probably 198 (36)	Unsure 28 (5)	Unlikely 19 (3)
Response if mHealth tool suggests that current condition is low risk	Visit Clinic 165 (30)	Use Telemedicine 62 (11)	Watchful Waiting* 152 (28)	Seek ED Care 170 (31)

*mHealth*, mobile health; *ED*, emergency department.

* Watchful waiting indicates that the patient was willing to manage symptoms at home with over-the-counter medications and later determine if medical attention was needed.
