# Supplementary material for: Potential of Mobile Health Technology to Reduce Health Disparities in Underserved Communities
Source: West J Emerg Med. 2019 Aug 6;20(5):799–802. doi: 10.5811/westjem.2019.6.41911 (PMC6754190; doi:10.5811/westjem.2019.6.41911)
Supplement: Supplementary file 1 [file wjem-20-799-s001.docx]

**Appendix 1**. Survey Instrument for Mobile Health Tool

1. Do you have a primary care doctor you can go to regularly? *Yes/No*
2. Do you have a doctor or nurse that you can call for medical advice? *Yes/No*
3. Do you use applications on your phone? *Yes/No*
4. Do you have access to the internet through a computer? *Yes/No*
5. Do you have access to the internet through your phone? *Yes/No*
6. Do you have access to the internet through another portable device? *Yes/No*
7. If you are uncertain about your health (or your child's health) do you contact a friend or family member? *Yes/No*
8. If you are uncertain about your health (or your child's health) do you call a doctor or a clinic nurse? *Yes/No*
9. If you are uncertain about your health (or your child's health) do you look up your condition in a book? *Yes/No*
10. If you are uncertain about your health (or your child's health) do you look online for advice? *Yes/No*
11. What websites do you use online for your health? *Free text response*
12. Would you use this proposed mobile health tool? *Definitely, Probably, Uncertain, or Unlikely*
13. If not, please describe why not. *Free text response*
14. If the proposed mobile health tool recommended that you (your child) has a low risk of a health emergency, which options would you choose?
    1. *Stay home, take recommended over the counter medications for symptoms, and reassess in 1-2 days if you need medical care (watchful waiting)*
    2. *Make a telemedicine visit (either video call or phone call with a clinician to assess your condition)*
    3. *See your primary care clinic as soon as possible*
    4. *Still visit the Emergency Department for your current condition*
15. What other suggestions do you have? *Free text response*
